# Supplementary material for: Identifying regions for enhanced control of gambiense sleeping sickness in the Democratic Republic of Congo
Source: Nat Commun. 2022 Mar 18;13:1448. doi: 10.1038/s41467-022-29192-w (PMC8933483; doi:10.1038/s41467-022-29192-w)
Supplement: Supplementary file 1 — Supplementary Information [file 41467_2022_29192_MOESM1_ESM.pdf]

Supplementary information: Identifying regions for enhanced control of *gambiense* sleeping sickness in the Democratic Republic of Congo

Ching-I Huang[1,2,+,*], Ronald E Crump[1,2,3,+], Paul E Brown[1,2], Simon E F Spencer[1,4], Erick Mwamba Miaka[5], Chansy Shampa[5], Matt J Keeling[1,2,3], and Kat S Rock[1,2]

[1]Zeeman Institute for System Biology and Infectious Disease Epidemiology Research, The University of Warwick, Coventry, U.K.
[2]Mathematics Institute, The University of Warwick, Coventry, U.K.
[3]The School of Life Sciences, The University of Warwick, Coventry, U.K.
[4]The Department of Statistics, The University of Warwick, Coventry, U.K.
[5]Programme National de Lutte contre la Trypanosomiase Humaine Africaine (PNLTHA), Kinshasa, D.R.C.
[*]ching-i.huang@warwick.ac.uk
[+]these authors contributed equally to this work

Supplementary Methods

This work is one of a series of studies on modelling and analyses on issues in the last mile of eliminating transmission of *gambiense* human African trypanosomiasis (gHAT) in the Democratic Republic of Congo (DRC). The series of studies starts with a model fitting paper[1] and to aid the reader of the present study, much of the same model information is provided here in Supplementary Methods.

1. The compartmental gHAT model

The gHAT model we considered in this study is a variant "Model 4" of the Warwick model presented in the literature[1–4]. gHAT infections among hosts are described by Supplementary Equation (1). Human hosts are modelled by the SEIIRS model with two infectious compartments, stage 1 disease, I_{1H}, and stage 2 disease, I_{2H}. Vectors are modelled by using compartments for appropriately modelling tsetse when used in a host-vector model with disease[3]. Pupal stage tsetse, P_V, emerge into unfed susceptible adults, S_V, and following a blood-meal become either exposed, E_V, or have reduced susceptibility to the *Trypanosoma brucei gambiense* parasites, G_V - this effect is known as the teneral phenomenon. Following an infection, tsetse have an extrinsic incubation period (EIP) before becoming onwardly infectious. To incorporate a more realistic EIP distribution, there are three exposed classes, E_{1V}, E_{2V}, E_{3V}, which result in a gamma-distributed EIP (rather than an exponential with only one).

In order to reduce the dimensionality of our ODE system (by one), the vector equations are non-dimensionalised using the scaling N_H/N_V, where N_H is the total human population, and N_V is the tsetse population size. This results in a new non-dimensionalised parameter, m_{eff}, which is $\dfrac{p_H N_V}{N_H}$ appearing in host equations (p_H is the probability of a human being infected by a single infectious bloodmeal) and is referred to as the *effective vector density*.

The proportion of tsetse bites taken on low-risk and high-risk humans are f_1 and f_4, depending on the relative availability/attractiveness and the relative abundance of two risk groups. High-risk humans are assumed to be r-fold more likely to receive bites, i.e. $s_1 = 1$ and $s_4 = r$. Therefore, f_i's can be calculated using $f_i = \dfrac{s_i N_{Hi}}{\sum_j s_j N_{Hj}}$.

$$
\text{Humans}\quad\begin{cases}
\dfrac{\mathrm{d}S_{Hi}}{\mathrm{d}t} &= \mu_H N_{Hi} + \omega_H R_{Hi} - \alpha m_{\mathrm{eff}} f_i \dfrac{S_{Hi}}{N_{Hi}} I_V - \mu_H S_{Hi} \\
\dfrac{\mathrm{d}E_{Hi}}{\mathrm{d}t} &= \alpha m_{\mathrm{eff}} f_i \dfrac{S_{Hi}}{N_{Hi}} I_V - (\sigma_H + \mu_H) E_{Hi} \\
\dfrac{\mathrm{d}I_{1Hi}}{\mathrm{d}t} &= \sigma_H E_{Hi} - (\varphi_H + \eta_H(Y) + \mu_H) I_{1Hi} \\
\dfrac{\mathrm{d}I_{2Hi}}{\mathrm{d}t} &= \varphi_H I_{1Hi} - (\gamma_H(Y) + \mu_H) I_{2Hi} \\
\dfrac{\mathrm{d}R_{Hi}}{\mathrm{d}t} &= \eta_H(Y) I_{1Hi} + \gamma_H(Y) I_{2Hi} - (\omega_H + \mu_H) R_{Hi}
\end{cases}
$$

$$
\text{Tsetse}\quad\begin{cases}
\dfrac{\mathrm{d}P_V}{\mathrm{d}t} &= B_V N_H - (\xi_V + \frac{P_V}{K}) P_V \\
\dfrac{\mathrm{d}S_V}{\mathrm{d}t} &= \xi_V \mathbb{P}(\text{survive pupal stage}) P_V - \alpha S_V - \mu_V S_V \\
\dfrac{\mathrm{d}E_{1V}}{\mathrm{d}t} &= \alpha\big(1 - f_T(t)\big) p_V \big(\sum_i f_i \dfrac{(I_{1Hi} + I_{2Hi})}{N_{Hi}} + f_A \dfrac{I_A}{N_A}\big)(S_V + \varepsilon G_V) \\
&\qquad -(3\sigma_V + \mu_V + \alpha f_T(t)) E_{1V} \\
\dfrac{\mathrm{d}E_{2V}}{\mathrm{d}t} &= 3\sigma_V E_{1V} - (3\sigma_V + \mu_V + \alpha f_T(t)) E_{2V} \\
\dfrac{\mathrm{d}E_{3V}}{\mathrm{d}t} &= 3\sigma_V E_{2V} - (3\sigma_V + \mu_V + \alpha f_T(t)) E_{3V} \\
\dfrac{\mathrm{d}I_V}{\mathrm{d}t} &= 3\sigma_V E_{3V} - (\mu_V + \alpha f_T(t)) I_V \\
\dfrac{\mathrm{d}G_V}{\mathrm{d}t} &= \alpha(1 - f_T(t)) S_V \\
&\qquad -\alpha\Big(f_T(t) + (1 - f_T(t)) p_V \varepsilon \big(\sum_i f_i \dfrac{(I_{1Hi} + I_{2Hi})}{N_{Hi}} + f_A \dfrac{I_A}{N_A}\big) G_V\big) \\
&\qquad\quad -\mu_V G_V
\end{cases}
\tag{1}
$$

2. Model assumptions and parameter estimates from MCMC fitting

Supplementary Table 1 provides the estimates of fixed parameters available in the literature used in the previous gHAT model[1–3]. The parameters fitted during the model fitting are defined in Supplementary Table 2. Posterior distributions for these parameters are estimated using MCMC methods[1] and are available in our graphical user interface at https://hatmepp.warwick.ac.uk/fitting/v2/, or can be downloaded in Open Science Framework at https://osf.io/ck3tr/.

Supplementary Table 1. Model parametrisation (fixed parameters). Notation, a brief description, and the used values for fixed parameters.

Notation	Description	Value	
N_H	Total human population size in 2015	Fixed for each health zone	[5]
μ_H	Natural human mortality rate	5.4795×10^{-5} days^{-1}	[6]
B_H	Total human birth rate	$= \mu_H N_H$	
σ_H	Human incubation rate	0.0833 days^{-1}	[7]
φ_H	Stage 1 to 2 progression rate	0.0019 days^{-1}	[8,9]
ω_H	Recovery rate or waning-immunity rate	0.006 days^{-1}	[10]
Sens	Active screening diagnostic sensitivity	0.91	[11]
B_V	Tsetse birth rate	0.0505 days^{-1}	[3]
ξ_V	Pupal death rate	0.037 days $^{-1}$	
K	Pupal carrying capacity	$= 111.09N_H$	[3]
$\mathbb{P}(\text{pupating})$	Probability of pupating	0.75	
μ_V	Tsetse mortality rate	0.03 days^{-1}	[7]
σ_V	Tsetse incubation rate	0.034 days^{-1}	[12,13]
α	Tsetse bite rate	0.333 days^{-1}	[14]
p_V	Probability of tsetse infection per infectious bite	0.065	[7]
ε	Reduced non-teneral susceptibility factor	0.05	[2]
f_H	Proportion of blood-meals on humans	0.09	[15]
disp_{act}	Overdispersion parameter for active detection	4×10^{-4}	[1]
$\text{disp}_{\text{pass}}$	Overdispersion parameter for passive detection	2.8×10^{-5}	[1]

[1] Value of B_V is chosen to maintain constant population size without interventions.
[2] Value of K is chosen to reflect the observed bounce back rate.

Supplementary Table 2. Model parametrisation (posteriors of fitted parameters). Notation, a brief description, and representative percentiles of the posterior distributions for fitted parameters.

Notation	Description	Posterior (median [95% CI]) Kwamouth	Tandala
R_0	Basic reproduction number (NGM approach)	1.09 [1.06, 1.14]	1.009 [1.006, 1.014]
r	Relative bites taken on high-risk humans	6.61 [3.15, 10.75]	2.04 [1.30, 4.26]
k_1	Proportion of low-risk people	0.90 [0.82, 0.95]	0.95 [0.85, 0.99]
γ_H^{pre}	Pre-1998 treatment rate from stage 2 (days^{-1})	1.72×10^{-3} $[0.38, 4.88] \times 10^{-3}$	2.53×10^{-3} $[1.03, 7.15] \times 10^{-3}$
η_H^{post}	Post-1998 treatment rate from stage 1 (days^{-1})	1.24×10^{-4} $[0.60, 2.74] \times 10^{-4}$	2.74×10^{-4} $[1.11, 4.99] \times 10^{-4}$
γ_H^{post}	Post-1998 treatment rate from stage 2 (days^{-1})	1.88×10^{-3} $[0.46, 5.42] \times 10^{-3}$	3.60×10^{-3} $[1.72, 8.98] \times 10^{-3}$
Spec	Active screening diagnostic specificity	0.9991 [0.9987, 0.9997]	0.9998 [0.9997, 0.9999]
u	Proportion of stage 2 passive cases reported	0.27 [0.18, 0.40]	0.39 [0.29, 0.51]
d_{change}	Midpoint year for passive improvement	2005.8 [2004.4, 2007.3]	–
$\eta_{H_{\text{amp}}}$	Relative improvement in passive stage 1 detection rate	2.52 [0.92, 5.46]	–
$\gamma_{H_{\text{amp}}}$	Relative improvement in passive stage 2 detection rate	0.51 [0.24, 0.97]	–
d_{steep}	Speed of improvement in passive detection rate (years^{-1})	0.94 [0.68, 1.29]	–

[1] See Supplementary Equation (2) for improved passive detections formulated by d_{change}, $\eta_{H_{\text{amp}}}$, $\gamma_{H_{\text{amp}}}$ and d_{steep}.

3. Active screening

Screening data is aggregated by year and the exact dates and frequencies of conducting active screening (AS) are unknown, therefore some assumptions were made on when AS takes place. Our model assumed only low-risk humans participate in AS and used the ratio of assumed number of people screened (N_{AS}) and the number of low-risk humans ($k_1 N_H$) to decide the frequency of AS each year. When screening numbers were smaller than potential participants ($N_{AS} < k_1 N_H$), a single AS event was assumed to take place at the beginning of those years. On the other hand, multiple AS events were evenly distributed over the time of the corresponding years, i.e. a second AS event in July when $k_1 N_H < N_{AS} \leq 2k_1 N_H$; a second AS event in May and a third AS event in September when $2k_1 N_H < N_{AS} \leq 3k_1 N_H$; etc.

4. Formulation and parametrisation of improved passive detections in Bandundu and Bas Congo

Previous analysis on provincial-level staged data (Lumbala *et al.* for 2000–2012[16] and WHO HAT Atlas data 2015–2016[17]) indicated that improved passive detection has happened across former Bandundu province and in former Bas Congo province[1]. Logistic functions shown in Supplementary Equation (2) were used to formulate the improved passive detections in Bandundu and Bas Congo in year Y.

$$\begin{aligned} \eta_H(Y) &= \eta_H^{\text{post}} \left[1 + \frac{\eta_{H_{\text{amp}}}}{1+\exp\left(-d_{\text{steep}}(Y - d_{\text{change}})\right)} \right], \\ \gamma_H(Y) &= \gamma_H^{\text{post}} \left[1 + \frac{\gamma_{H_{\text{amp}}}}{1+\exp\left(-d_{\text{steep}}(Y - d_{\text{change}})\right)} \right]. \end{aligned} \tag{2}$$

Parameter definitions and their posteriors are provided in Supplementary Table 2. N.B. It was assumed that improvements in both stages shared the same midpoint year and speed of improvement within a health zone. However, the amplitude of variation in each health zone came from the fitting of health-zone-specific data.

5. Formulation and parametrisation of additional tsetse mortality under vector control measures

The function which describes the probability of both hitting a target and dying is time dependent (days) from when the targets where placed:

$$f_T(t) = f_{\text{max}} \left(1 - \frac{1}{1+\exp(-0.068(\text{mod}(t, 182.5) - 127.75))} \right), \tag{3}$$

and f_{max} is chosen such that the tsetse population after one year is at the observed/assumed percentage reduction. For the simplified model this is given by $f_{\text{max}} = 0.0305$ for a 60% reduction, $f_{\text{max}} = 0.0525$ for an 80% reduction, and $f_{\text{max}} = 0.0750$ for a 90% reduction.

6. Simulations performed

Simulations were performed based on 1,000 model realisations. Observation uncertainty was considered by drawing ten random samples from the predicted mean dynamics for each set of parameters. A beta-binomial distribution in which an overdispersion parameter ρ was introduced to the binomial distribution was used to account for larger variance than the binomial. The probability of obtaining m successes out of n trials with probability p and overdispersion parameter ρ is

$$\text{BetaBin}(m; n, p, \rho) = \frac{\Gamma(n+1)\Gamma(m+a)\Gamma(n-m+b)\Gamma(a+b)}{\Gamma(n-m+1)\Gamma(n+a+b)\Gamma(a)\Gamma(b)}, \tag{4}$$

where $a = p(1/\rho - 1)$ and $b = a(1-p)/p$.

Main observable outputs including active and passive cases each year were predicted by 10,000 samples. Unobservable outputs, such as new infections and the year of elimination of transmission, were predicted directly from the 1,000 model realisations without sampling (parameter uncertainty but no observation uncertainty). Our model also has the capability of outputting unreported deaths and person years spent in stage 1 and stage 2.

7. Uncertainty

Model predictions propagate parameter and observation uncertainty (see above). We tried to represent this uncertainty in a variety of ways:

- Time series box plots were used to display statistical summaries of model predictions – the median (the middle line in each box), the lower and upper quartiles (the edges of each box showing 50% prediction intervals) and 95% prediction intervals (extended whiskers containing the middle 95% of outputs).
- The median year of elimination of transmission was used to indicate the estimated elimination year for a series of model predictions because neither extreme values (outliers) nor truncation of simulation will affect the estimates.
- Probability of elimination of transmission (EOT) by 2030 (or any given year between 2020 and 2040 in the graphical user interface at `https://hatmepp.warwick.ac.uk/projections/v2/`) was calculated as the proportion of model realisations that achieve EOT by 2030 or the given year. Its continuous spectrum shows how likely we are to achieve EOT by a particular year and highlights regions we are most uncertain about with respect to meeting this goal.
- Sensitivity analysis for vector control (VC) was performed to provide additional assessment of the VC reduction assumption (see results below).

8. Proxy for elimination of transmission

The gHAT model we used here is a deterministic model described by ODEs with transition rates between compartments. In the deterministic model, variables such as new infections, new cases and deaths can be non-integer and their values are continuous. The stochastic model, on the other hand, has dynamics driven by randomly occurring events with associated probabilities and its variables capture the discrete nature of the population. Despite good agreements on the mean dynamics in both models, even at very low prevalence, the dynamics at the endgame are different. Because of the continuous nature of deterministic dynamics, the number of infected people asymptotes to zero rather than reaching it unlike the stochastic model. In this paper, an artificial elimination of transmission (EOT) threshold i.e. one new infection per health zone per year was applied to new infections to determine whether EOT has been achieved or not. Other values of EOT thresholds, such as one new infection per 100,000 or per 1,000,000 people per year, can be found in the literature[18,19]. Large variation in EOT threshold highlights the difficulty in choosing a proper threshold reflecting the reality. More detailed comparison between stochastic and deterministic model variants will be needed in the future to ensure robustness of year of EOT estimates arising from such a proxy threshold.

Supplementary Note 1: Model Updates

Previously published variations on this "Warwick gHAT model" are described in Supplementary Methods. Key differences between this model and previous versions (notably[19]) have been described in the study when the model was fitted to data[1] and are listed again here:

- Improved passive detection: Improvement due to the introduction of CATT test ($\eta_H^{\text{pre}} = 0 \mapsto \eta_H^{\text{post}}$ and $\gamma_H^{\text{pre}} = b_{\gamma_H^{\text{pre}}} \gamma_H^{\text{post}} \mapsto \gamma_H^{\text{post}}$ in Crump *et al*[1]) is considered in 1998 in the entire DRC as well as gradual improvements of stage 1 and stage 2 passive detection rates over time in Supplementary Equation (2) are taken into account in Bandundu and Bas Congo provinces.
- MSF active screening algorithm: The results of CATT 1:32, which has a higher sensitivity (MSF sensitivity = 0.95 in contrast to PNLTHA sensitivity = 0.91) and a lower fitted specificity ($= b_{\text{specificity}} \times$ specificity with targeted mean = 0.991 in Crump *et al*[1]) than the PNLTHA-DRC algorithm, were used for case reporting and treatments in Orientale province in active screenings performed by MSF before 2013.
- Perfect specificity in active screening: Video confirmation was introduced in Mosango and Yasa Bonga since 2015 and therefore the specificity in active screenings became 1 since then. Perfect specificity is also assumed in the rest of Bandundu from 2018. The diagnostic algorithms automatically change to 100% specificity outside Bandundu when the detected case numbers were close to the expected incidence of false positive detections.
- Overdispersion in case detections: Observation uncertainty is considered by adding overdispersion into case detections in Supplementary Equation (4). To avoid overfitting, the overdispersion parameters were manually tuned to be appropriate for a health zone level fit, and thereafter left fixed across MCMC runs in Crump *et al*[1].
- Elimination of transmission (EOT) threshold: A proxy threshold (= 1) of new infection per health zone per year is used to identify when EOT has been reached within this deterministic framework. Previous versions of this model have used a variety of proxy thresholds, typically fewer than one new infection per 100,000 per year[18,19].
- No transmission after achieving EOT: Unlike previous Warwick gHAT model variants, once the EOT threshold is met we set transmission to zero in subsequent years and therefore no further new infections is possible. Previously infected people can still be identified and reported (although there are typically extremely few reported cases at that point).

Supplementary Note 2: Results

1. Trends under strategies with historical maximum active screening (HistMaxAS and HistMaxAS+VC)

The historical maximum active screening (AS) coverage between 2000–2016 represents maximum level of resource that each health zone has previously received and increasing coverage beyond this may require not only additional funding but also capacity building. According to the WHO HAT Atlas data, the median of the maximum coverage is 10.56%. The highest record, 82.08%, was in 2010 from the Bolobo health zone of the former Bandundu province. PNLTHA-DRC screened nearly 1.6 million people in the entire country and over 100,000 in Bolobo health zone that year. Supplementary Fig. 1 shows the projections of active and passive cases, new infections, and probability of elimination of transmission (EOT) under two strategies with historical AS maximum coverage (HistMaxAS and HistMaxAS+VC) in our two example health zones: Kwamouth and Tandala. The historical maximum AS coverages are 73.42% in Kwamouth and 34.97% in Tandala. The AS coverage in the HistMaxAS(+VC) strategies in Tandala is slightly lower than 40%, the model outputs are very similar to those under the 40%AS(+VC) strategies (Figure 1 in the main text). For Kwamouth, EOT is still very unlikely under the HistMaxAS strategy despite its high AS coverage.

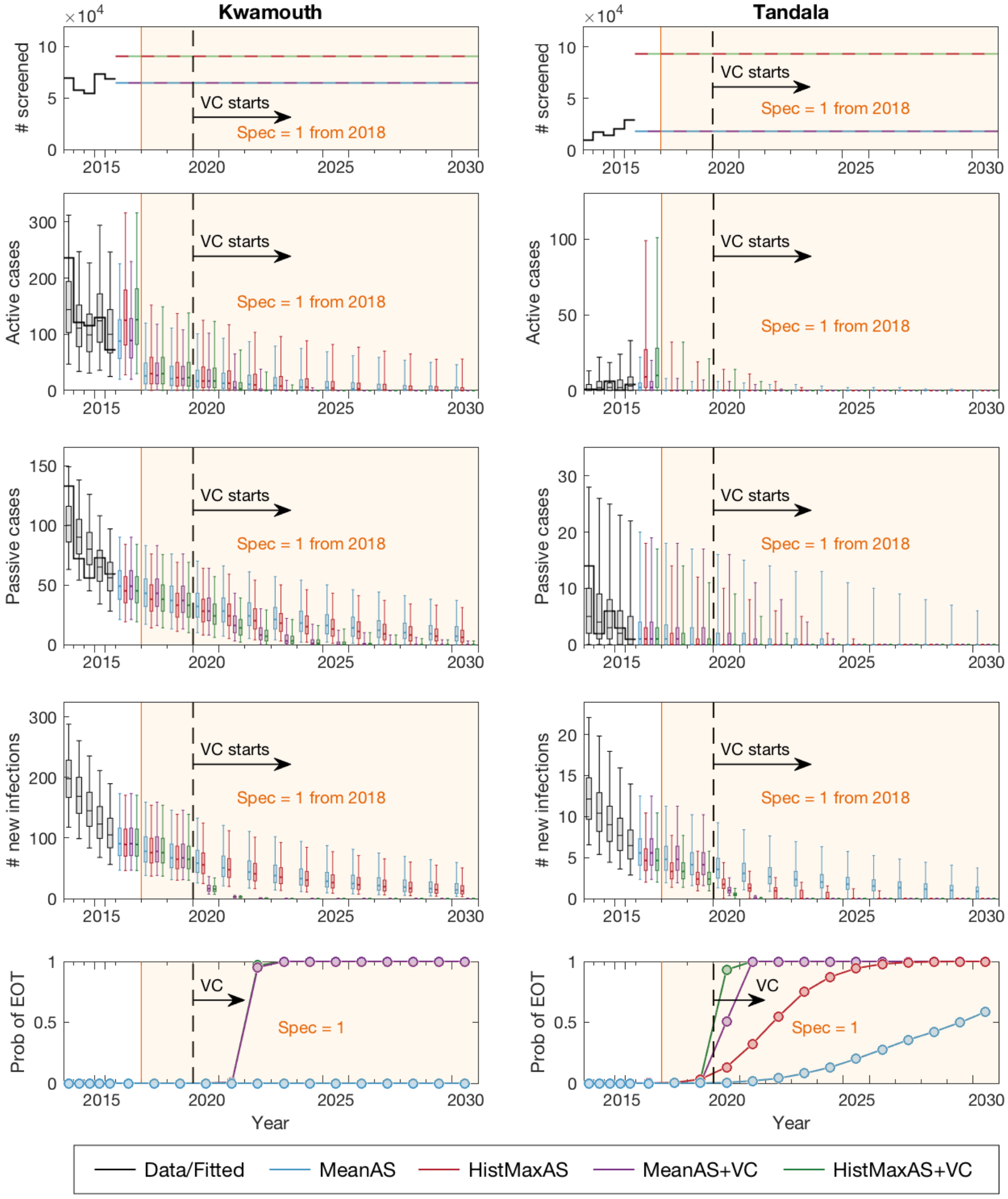

Supplementary Fig. 1. Projections under the HistMaxAS and HistMaxAS+VC strategies. There are n = 10,000 independent samples, 10 from each of 1,000 independent samples from the joint posterior distributions of the fitted model parameters. Box plots summarise parameter and observational uncertainty. The lines in the boxes present the medians of predicted results. The lower and upper bounds of the boxes indicate 25th and 75th percentiles. The minimum and maximum values are 2.5th and 97.5th percentiles and therefore whiskers cover 95% prediction intervals. Full model outputs (2000–2050) of all 168 analysed health zones are available in the graphical user interface at https://hatmepp.warwick.ac.uk/projections/v2/.

AS: active screening; VC: vector control; Hist: historical; Spec: specificity; EOT: elimination of transmission

2. Sensitivity analysis of the effectiveness of vector control

The reported annual reductions in tsetse populations of vector control (VC) range from 80% to 99%[4,20–22]. The reductions are highly variable between locations due to differences in accessibility and the effectiveness of target deployments. In addition to the default tsetse reduction assumed here (80%), a conservative and a high but achievable reduction, 60% and 90%, are considered in the sensitivity analysis of VC. As shown in Supplementary Fig. 2, the sensitivity analysis shows that the effectiveness of VC has a direct impact on the numbers of underlying new infections and leading to a delay to elimination of transmission when VC effectiveness is low.

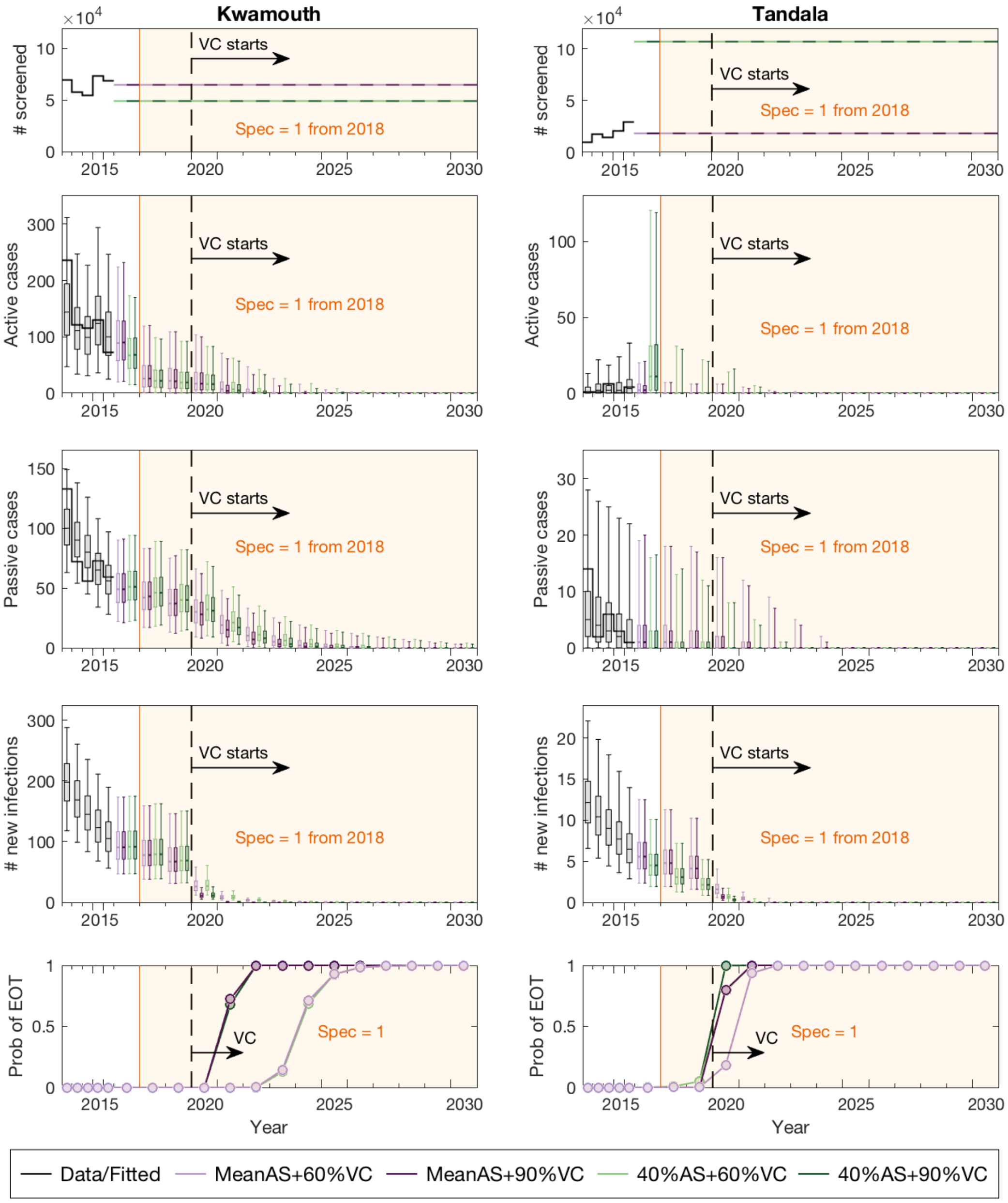

Supplementary Fig. 2. Sensitivity analysis of vector control. There are n = 10,000 independent samples, 10 from each of 1,000 independent samples from the joint posterior distributions of the fitted model parameters. Box plots summarise parameter and observational uncertainty. The lines in the boxes present the medians of predicted results. The lower and upper bounds of the boxes indicate 25th and 75th percentiles. The minimum and maximum values are 2.5th and 97.5th percentiles and therefore whiskers cover 95% prediction intervals. Full model outputs (2000–2050) of all 168 analysed health zones are available in the graphical user interface at `https://hatmepp.warwick.ac.uk/projections/v2/`.
AS: active screening; VC: vector control; Spec: specificity; EOT: elimination of transmission

3. Total deaths under the MeanAS strategy and deaths averted with intensified interventions

The disability-adjusted life year (DALY) is a common measure of disease burden for low- and middle-income countries. Sleeping sickness is a fatal disease if untreated, therefore death is the dominant term in its DALYs. Supplementary Fig. 3 shows the estimated total deaths from 2017–2030 under the MeanAS strategy in each health zone. The former provinces of Bandundu, Bas Congo and Equateur had the highest disease burden in the early 2000s. Our model predicts very few total deaths in Bas Congo and Equateur provinces if interventions are maintained at the current level. But Bandundu province still has high disease burden despite its recent advances in passive screening and high coverage in active screening.

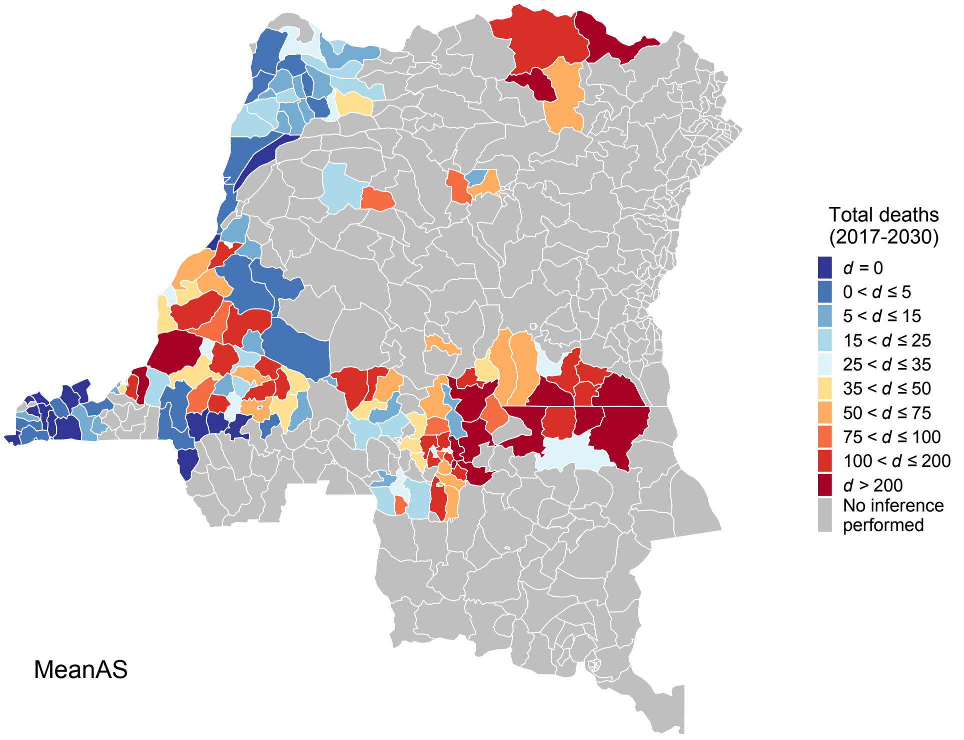

Supplementary Fig. 3. Health zone total death map (2017–2030) under the MeanAS strategy. Shapefiles used to produce this map are available under an ODC-ODbL licence at `https://data.humdata.org/dataset/drc-health-data`.
AS: active screening

Deaths averted when intensified interventions are implemented are defined as the difference in deaths between the MeanAS strategy and the intensified strategy. Supplementary Fig. 4 shows predicted deaths averted under four strategies (HistMaxAS, 40%AS, MeanAS+VC, and 40%AS+VC). Only a few health zones have high deaths averted under the HistMaxAS strategy because (1) the historical maximum AS coverage is too low to pick up many infected individuals in some health zones, particularly in the east of the country; (2) in health zones where the mean AS coverage is already good (e.g. in former Bandundu province), there can be marginal benefit from increasing coverage as our simulated strategy never reaches high-risk individuals through AS. By comparing deaths averted under the 40%AS strategy to that under the HistMaxAS strategy, large numbers of deaths are predicted to be averted in Kasai Occidental, Kasai Oriental, Katanga and Maniema provinces. Note that there are two health zones, Kwamouth and Masi Manimba in the former Bandundu province, having negative values in deaths averted under the 40%AS strategy because their mean AS coverage is higher than 40%. Despite high efficacy of AS in reducing disease burden, our model predicts the MeanAS+VC strategy averts more deaths in the former Bandundu province than medical only strategies. It suggests that transmission is still very active and VC is highly recommended in these health zones. Finally, the 40%AS+VC strategy, which has the same chance of achieving EOT by 2030 (in the main text), prevents more deaths compared to the MeanAS+VC strategy.

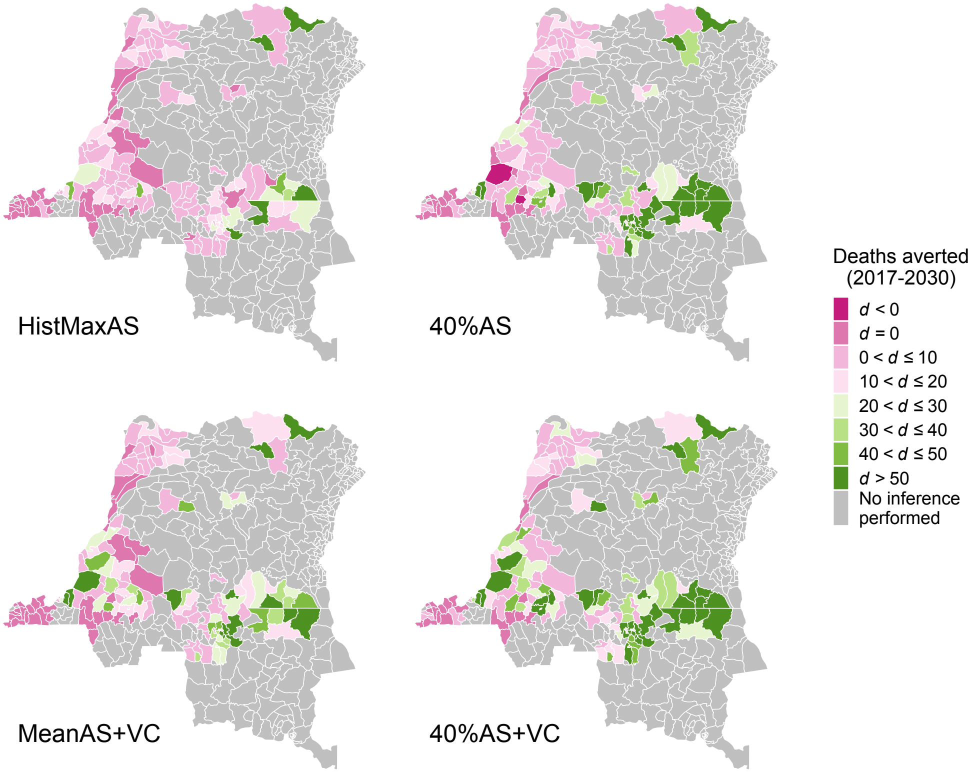

Supplementary Fig. 4. Health zone deaths averted maps for intensified strategies. Shapefiles used to produce maps are available under an ODC-ODbL licence at https://data.humdata.org/dataset/drc-health-data.
AS: active screening; VC: vector control; Hist: historical

Supplementary Note 3: PRIME-NTD criteria

It has been recommended that good modelling practises should meet the five key principles relating to communication, quality and relevance of analyses – known as Policy-Relevant Items for Reporting Models in Epidemiology of Neglected Tropical Diseases (PRIME-NTD)[23]. We present how these PRIME-NTD criteria have each been addressed in Supplementary Table 3.

Principle and what has been done to satisfy the principle?	Where in the manuscript is this described?
**1. Stakeholder engagement**<br>This study was lead by modellers and guided by members of the national sleeping sickness control programme in DRC (PNLTHA-DRC) – coauthors Erick Mwamba Miaka and Chansy Shampa. PNLTHA-DRC have contributed to improved modeller understanding of the epidemiological data and changes to the programme over time and in different geographic regions, both of which impacted model fitting over several rounds of revision (via in-person meetings and email). The GUI (and several variants of it) was designed in conjunction with PNLTHA-DRC to improve communication of the modelling outputs to non-modellers. It has been refined through in-person meetings with collaborators from multiple institutions with the goal of providing understandable, policy-relevant outputs combined with scientific communication; over 20 non-modellers have had opportunities to interact with and provide feedback on the GUI during development.	Authorship list
**2. Complete model documentation**<br>The full model (including the fitting code) and documentation are available in OSF. The model is fully described in Methods and Supplementary Methods and in the fitting study of Crump *et al*[1].	Methods, Supplementary Methods, and OSF (for Matlab code) at https://osf.io/jza27/
**3. Complete description of data used**<br>The data used for fitting were described in Crump *et al*[1]. Posteriors used for the projections in this paper are available in OSF.	OSF (for posteriors) at https://osf.io/ck3tr/
**4. Communicating uncertainty**<br>*Structural uncertainty*:<br>The variant of the model presented here ("Model 4") was chosen as it had good support compared to other plausible model structures when fitting to data sets from Yasa Bonga and Mosango health zones in DRC[2] and in the Mandoul focus, Chad[4].	Methods
Parameter uncertainty:<br>In the fitting study[1] key model parameters were fitted in an MCMC framework by utilising regional data, we used these posterior parameter sets to simulate forward projections in the present study. Sensitivity analysis of VC was performed on the assumed impact of tsetse reductions (60% and 90%) in addition to the default assumption of 80% reduction.	Methods, Crump *et al*[1], OSF (for posteriors) at https://osf.io/ck3tr/, Supplementary Fig. 2 (for sensitivity analysis of VC), and the GUI at https://hatmepp.warwick.ac.uk/projections/v2/
Prediction uncertainty:<br>Here, we represent uncertainty in our results in various ways - (i) by providing box and whisker plots for predictions (median, 50% and 95% prediction intervals), (ii) by utilising probability maps (likelihood of meeting EOT goal by 2030) in addition to median year of EOT maps, and (iii) by providing prediction intervals for EOT years in the hoover feature in our GUI.	Figures 1 and 3, Supplementary Fig. 1, and maps in the GUI at https://hatmepp.warwick.ac.uk/projections/v2/
**5. Testable model outcomes**<br>Predictions presented here include measurable and routinely reported outcomes, such as active and passive case reporting by year. Outputs in the GUI can be compared to new case data as it becomes available. Our predictions are dependant on the coverage of active screening each year and are expected to perform better when the actual screening coverage is similar to that assumed for predicting. In the future we plan to validate the predictions based on at least two years of new case data to assess model performance.	Figure 1, Supplementary Fig. 1, the GUI at https://hatmepp.warwick.ac.uk/projections/v2/, and OSF (for model code and posteriors) at https://osf.io/jza27/ and https://osf.io/ck3tr/

Supplementary Table 3. PRIME-NTD criteria fulfillment. We summarise how the NTD Modelling Consortium's "5 key principles of good modelling practice" have been met in the present study.
GUI: graphical user interface; OSF: Open Science Framework; VC: vector control; EOT: elimination of transmission

Supplementary References

1. Crump, R. E. *et al.* Quantifying epidemiological drivers of *gambiense* human African trypanosomiasis across the Democratic Republic of Congo. *PLOS Comput. Biol.* **17**, 1–23 (2021).
2. Rock, K. S., Torr, S. J., Lumbala, C. & Keeling, M. J. Quantitative evaluation of the strategy to eliminate human African trypanosomiasis in the Democratic Republic of Congo. *Parasites & Vectors* **8**, 532 (2015).
3. Rock, K. S., Torr, S. J., Lumbala, C. & Keeling, M. J. Predicting the impact of intervention strategies for sleeping sickness in two high-endemicity health zones of the Democratic Republic of Congo. *PLOS Neglected Trop. Dis.* **11**, e0005162 (2017).
4. Mahamat, M. H. *et al.* Adding tsetse control to medical activities contributes to decreasing transmission of sleeping sickness in the Mandoul focus (Chad). *PLOS Neglected Trop. Dis.* **11**, e0005792 (2017).
5. OCHA Office for the Coordination of Humanitarian Affairs. Journees Nationales de Vaccination (JNV) activities de vaccination supplementaire, RDC. https://data.humdata.org/dataset/rdc-statistiques-des-populations. (accessed May 2016).
6. The World Bank. Data: Democratic Republic of Congo. https://data.worldbank.org/country/congo-dem-rep?view=chart. (accessed 2015).
7. Rogers, D. J. A general model for the African trypanosomiases. *Parasitology* **97**, 193–212 (1988).
8. Checchi, F., Filipe, J. A. N., Barrett, M. P. & Chandramohan, D. The natural progression of *gambiense* sleeping sickness: what is the evidence? *PLOS Neglected Trop. Dis.* **2**, e303 (2008).
9. Checchi, F., Funk, S., Chandramohan, D., Haydon, D. T. & Chappuis, F. Updated estimate of the duration of the meningo-encephalitic stage in *gambiense* human African trypanosomiasis. *BMC Res. Notes* **8**, 292 (2015).
10. Mpanya, A. *et al.* Should I get screened for sleeping sickness? A qualitative study in Kasai province, Democratic Republic of Congo. *PLOS Neglected Trop. Dis.* **6**, e1467 (2012).
11. Checchi, F., Chappuis, F., Karunakara, U., Priotto, G. & Chandramohan, D. Accuracy of five algorithms to diagnose *gambiense* human African trypanosomiasis. *PLOS Neglected Trop. Dis.* **5**, e1233 (2011).
12. Davis, S., Aksoy, S. & Galvani, A. P. A global sensitivity analysis for African sleeping sickness. *Parasitology* **138**, 516–526 (2010).
13. Ravel, S., Grebaut, P., Cuisance, D. & Cuny, G. Monitoring the developmental status of *Trypanosoma brucei gambiense* in the tsetse fly by means of PCR analysis of anal and saliva drops. *Acta Trop.* **88**, 161–165 (2003).
14. World Health Organization & WHO Expert Committee on the Control and Surveillance of Human African Trypanosomiasis (2013: Geneva, Switzerland). *Control and surveillance of human African trypanosomiasis: report of a WHO expert committee*. WHO technical report series; no. 984 (World Health Organization, 2013).
15. Clausen, P.-H. *et al.* Host preferences of tsetse (Diptera: Glossinidae) based on bloodmeal identifications. *Med. Vet. Entomol.* **12**, 169–180 (1998).
16. Lumbala, C. *et al.* Human African trypanosomiasis in the Democratic Republic of the Congo: disease distribution and risk. *Int. J. Heal. Geogr.* **14**, 20 (2015).
17. Simarro, P. P., Cecchi, G. & Jannin, J. G. The Atlas of human African trypanosomiasis: a contribution to global mapping of neglected tropical diseases. *Int. J. Heal. Geogr.* **9**, 57 (2010).
18. Rock, K. S. *et al.* Assessing strategies against *gambiense* sleeping sickness through mathematical modeling. *Clin. Infect. Dis.* **66**, S286–S292 (2018).
19. Castaño, M. S. *et al.* Assessing the impact of aggregating disease stage data in model predictions of human African trypanosomiasis transmission and control activities in Bandundu province (DRC). *PLOS Neglected Trop. Dis.* **14**, e0007976 (2020).
20. Courtin, F. *et al.* Reducing human-tsetse contact significantly enhances the efficacy of sleeping sickness active screening campaigns: a promising result in the context of elimination. *PLOS Neglected Trop. Dis.* **9**, e0003727 (2015).
21. Tirados, I. *et al.* Tsetse control and gambian sleeping sickness; implications for control strategy. *PLOS Neglected Trop. Dis.* **9**, 1–22 (2015).

22. Tirados, I. *et al.* Impact of tiny targets on *Glossina fuscipes quanzensis*, the primary vector of human African trypanosomiasis in the Democratic Republic of the Congo. *PLOS Neglected Trop. Dis.* **14**, 1–20 (2020).

23. Behrend, M. R. *et al.* Modelling for policy: The five principles of the Neglected Tropical Diseases Modelling Consortium. *PLOS Neglected Trop. Dis.* **14**, e0008033 (2020).
