# Supplementary material for: Identifying regions for enhanced control of gambiense sleeping sickness in the Democratic Republic of Congo
Source: Nat Commun. 2022 Mar 18;13:1448. doi: 10.1038/s41467-022-29192-w (PMC8933483; doi:10.1038/s41467-022-29192-w)
Supplement: Supplementary file 3 — Reporting Summary [file 41467_2022_29192_MOESM3_ESM.pdf]

nature research

Corresponding author(s): Ching-I Huang

Last updated by author(s): Feb 4, 2022

Reporting Summary

Nature Research wishes to improve the reproducibility of the work that we publish. This form provides structure for consistency and transparency in reporting. For further information on Nature Research policies, see our Editorial Policies and the Editorial Policy Checklist.

Statistics

For all statistical analyses, confirm that the following items are present in the figure legend, table legend, main text, or Methods section.

n/a	Confirmed	
☒	☐	The exact sample size (*n*) for each experimental group/condition, given as a discrete number and unit of measurement
☒	☐	A statement on whether measurements were taken from distinct samples or whether the same sample was measured repeatedly
☒	☐	The statistical test(s) used AND whether they are one- or two-sided<br>*Only common tests should be described solely by name; describe more complex techniques in the Methods section.*
☒	☐	A description of all covariates tested
☒	☐	A description of any assumptions or corrections, such as tests of normality and adjustment for multiple comparisons
☐	☒	A full description of the statistical parameters including central tendency (e.g. means) or other basic estimates (e.g. regression coefficient) AND variation (e.g. standard deviation) or associated estimates of uncertainty (e.g. confidence intervals)
☒	☐	For null hypothesis testing, the test statistic (e.g. *F*, *t*, *r*) with confidence intervals, effect sizes, degrees of freedom and *P* value noted<br>*Give P values as exact values whenever suitable.*
☒	☐	For Bayesian analysis, information on the choice of priors and Markov chain Monte Carlo settings
☒	☐	For hierarchical and complex designs, identification of the appropriate level for tests and full reporting of outcomes
☒	☐	Estimates of effect sizes (e.g. Cohen's *d*, Pearson's *r*), indicating how they were calculated

Our web collection on statistics for biologists contains articles on many of the points above.

Software and code

Policy information about availability of computer code

Data collection	We are not resposible for data collection.
Data analysis	We used MATLAB R2018b for performing simulations and the entire code is available from: https://osf.io/jza27/

For manuscripts utilizing custom algorithms or software that are central to the research but not yet described in published literature, software must be made available to editors and reviewers. We strongly encourage code deposition in a community repository (e.g. GitHub). See the Nature Research guidelines for submitting code & software for further information.

Data

Policy information about availability of data

All manuscripts must include a data availability statement. This statement should provide the following information, where applicable:

- Accession codes, unique identifiers, or web links for publicly available datasets
- A list of figures that have associated raw data
- A description of any restrictions on data availability

Case data used for model fitting in Crump et al. and screening data used to inform future potential screening coverage were obtained through the World Health Organisation (WHO) HAT Atlas. Identifiable data cannot be shared publicly because of our data-sharing agreement with the WHO's HAT Atlas which is under the stewardship of the WHO. Data are available from the WHO (contact neglected.diseases@who.int or visit https://www.who.int/trypanosomiasis_african/country/foci_AFRO/en/) for researchers who meet the criteria for accessing confidential data. Time-frame for response to requests would depend on the WHOs schedule.

Field-specific reporting

Please select the one below that is the best fit for your research. If you are not sure, read the appropriate sections before making your selection.

☒ Life sciences ☐ Behavioural & social sciences ☐ Ecological, evolutionary & environmental sciences

For a reference copy of the document with all sections, see nature.com/documents/nr-reporting-summary-flat.pdf

Life sciences study design

All studies must disclose on these points even when the disclosure is negative.

Sample size	As this is a simulation study, there is no sample size.
Data exclusions	As this is a simulation study, there are no data exclusions.
Replication	As this is a simulation study, there was no replicability of experiments, but Monte Carlo samples were designated to be large enough to yield stable results.
Randomization	The study does not involve the allocation of patients or resources to treatments, and hence there is no randomisation in that sense. However, as an in silico simulation study, there is randomisation in the sense that random samples are drawn around the expected outcomes.
Blinding	This is not an experimental study, and so there are no trial participants to be blinded. The data to which the models were fitted (the subject of a previous article) were case records collectedby screening teams or in medical facilities.

Reporting for specific materials, systems and methods

We require information from authors about some types of materials, experimental systems and methods used in many studies. Here, indicate whether each material, system or method listed is relevant to your study. If you are not sure if a list item applies to your research, read the appropriate section before selecting a response.

Materials & experimental systems

n/a	Involved in the study	
☒	☐	Antibodies
☒	☐	Eukaryotic cell lines
☒	☐	Palaeontology and archaeology
☒	☐	Animals and other organisms
☒	☐	Human research participants
☒	☐	Clinical data
☒	☐	Dual use research of concern

Methods

n/a	Involved in the study	
☒	☐	ChIP-seq
☒	☐	Flow cytometry
☒	☐	MRI-based neuroimaging
